# Influence of Vocalized Reading Practice on English Learning and Psychological Problems of Middle School Students

**DOI:** 10.3389/fpsyg.2021.709023

**Published:** 2021-10-18

**Authors:** Hongyan Zhang, Xianghua Han

**Affiliations:** ^1^Second Language Acquisition, Translation Theory and Practice, Zhoukou Normal University, ZhouKou, China; ^2^Second Language Acquisition, Henan Finance University, Zhengzhou, China

**Keywords:** educational psychology, reading aloud training, self-efficacy, mental disorder in english learning, english learning anxiety

## Abstract

The purpose of this study is to improve the English learning anxiety and learning effect for middle school students. From the perspective of educational psychology, the influence of vocalized reading practice on the English learning of students is studied based on the self-efficacy theory and the schema theory. To encourage the students to practice English, the study might solve the problem of insufficient opportunities by applying the artificial intelligence (AI) chat system to the oral English practice of the students. Several research hypotheses are put forward, which concern the correlation between the English learning anxiety of the students with their self-efficacy, topic familiarity, and English grades under vocalized reading practice. Then, the hypotheses are verified through a controlled trial and a questionnaire survey (QS). Afterward, the experimental and QS data are statistically analyzed and tested with a regression model. The results show that the English grades, self-efficacy, and topic familiarity of the students have been significantly improved in the experimental group after the vocalized reading practice. The significance coefficient of the regression model is *P* = 0.000 < 0.05, which can be used to verify the proposed hypotheses. The English grades, self-efficacy, and topic familiarity can well-predict the English learning anxiety of the students. The computer simulation in educational communication (CSIEC) teaching system and AI can help create an interactive learning environment for the students to practice oral English by chatting with AI robots.

## Introduction

In China, the psychological problems of the students have not been paid enough attention in the current educational system, while too much emphasis has been on the development of the cognitive ability of the students, thus giving rise to the so-called “psychological crises” in education (Jacobson, 2020). With the evolution of educational psychology, the psychological problems of students in language learning are concerning more scholars in the educational circles and have been attached to great importance (McCrudden and Marchand, 2020). In particular, it has been argued that positive psychology is conducive to language learning, while negative psychology or psychological problems, hinders language learning. Specifically, positive psychology can enhance the positive emotional experiences of the students and cultivate healthy personality quality; meanwhile, it can help create a more effective learning atmosphere; moreover, it encourages the students to develop a positive outlook on life. It has been contended that anxiety can be the biggest psychological obstacle in learning a foreign language. For example, studies have revealed that anxiety in language learning affects the effect of language storage and output (Gumartifa and Syahri, 2021).

The vocalized reading practice has always been a popular teaching method to cultivate the sense of a language of students. In most cases, the students are impossible to learn a second language in its native linguistic context, so the vocalized reading practice becomes essential for the beginners to establish a speech signal system for a second language (Lou and Noels, 2020) in a traditional teaching method. In China, under the examination-oriented education evaluation system, most teachers and students are reluctant to spend time in the vocalized English reading practice. However, the vocalized reading practice can improve the oral English of the students, encourage them to communicate with English, and enjoy the charm of English rather than only learning it for the sake of examinations. Thereby, the interest of students in English can be aroused, as well as self-confidence in learning and speaking and English scores (Görgen et al., 2020). In terms of teaching, the English teachers should inspire the students to improve their oral English expressions and communications more through the vocalized reading practice. The application of artificial intelligence (AI) in English teaching can encourage students to chat in English, devise a dialog, and practice it with the robots in simulated environments. The AI robot provides learners with a simulated intelligent English context through individualized, diversified, and efficient methods to promote their autonomous learning.

The students with difficulties in English learning often lack self-confidence, have low interest in learning, and are easy to get cold feet in learning, so they are badly in need of help. This study aimed to improve the English learning effect of the middle school students and help them overcome the psychological problems in English learning. Section Literature review reviews the literature research on English learning anxiety and learning self-efficacy of the students; section Research methods and models puts forward the relevant hypotheses according to the existing research conclusions and explains the research methods and processes; section Results and Discussion lists and explains the actual investigation and analysis results, and finally, draws the research conclusion. Based on educational psychology, the vocalized reading practice in English learning is discussed, and its influence on relieving the English learning anxiety of the students is studied according to the self-efficacy theory and the schema theory. The research hypotheses are verified by a control experiment and a questionnaire survey (QS). Then, the experimental and QS data are statistically analyzed and tested through a regression model to explore the influence of self-efficacy, topic familiarity, and English scores on relieving the English learning anxiety of the students. Finally, the effect of vocalized reading practice is verified through different training results of the experimental class and the control class.

## Literature Review

### English Learning Anxiety

An American psychologist Horwitz first proposes the concept of “foreign language classroom anxiety” and defines it as “a kind of self-perception, belief, and emotion related to language learning in the class.” Horwitz finds that the students with foreign language classroom anxiety have less intention to speak in class, and they are nervous or afraid, often accompanied by a faster heartbeat, sweating, and other psychological problems (Horwitz and Albert, 2006).

**Figure 1:**
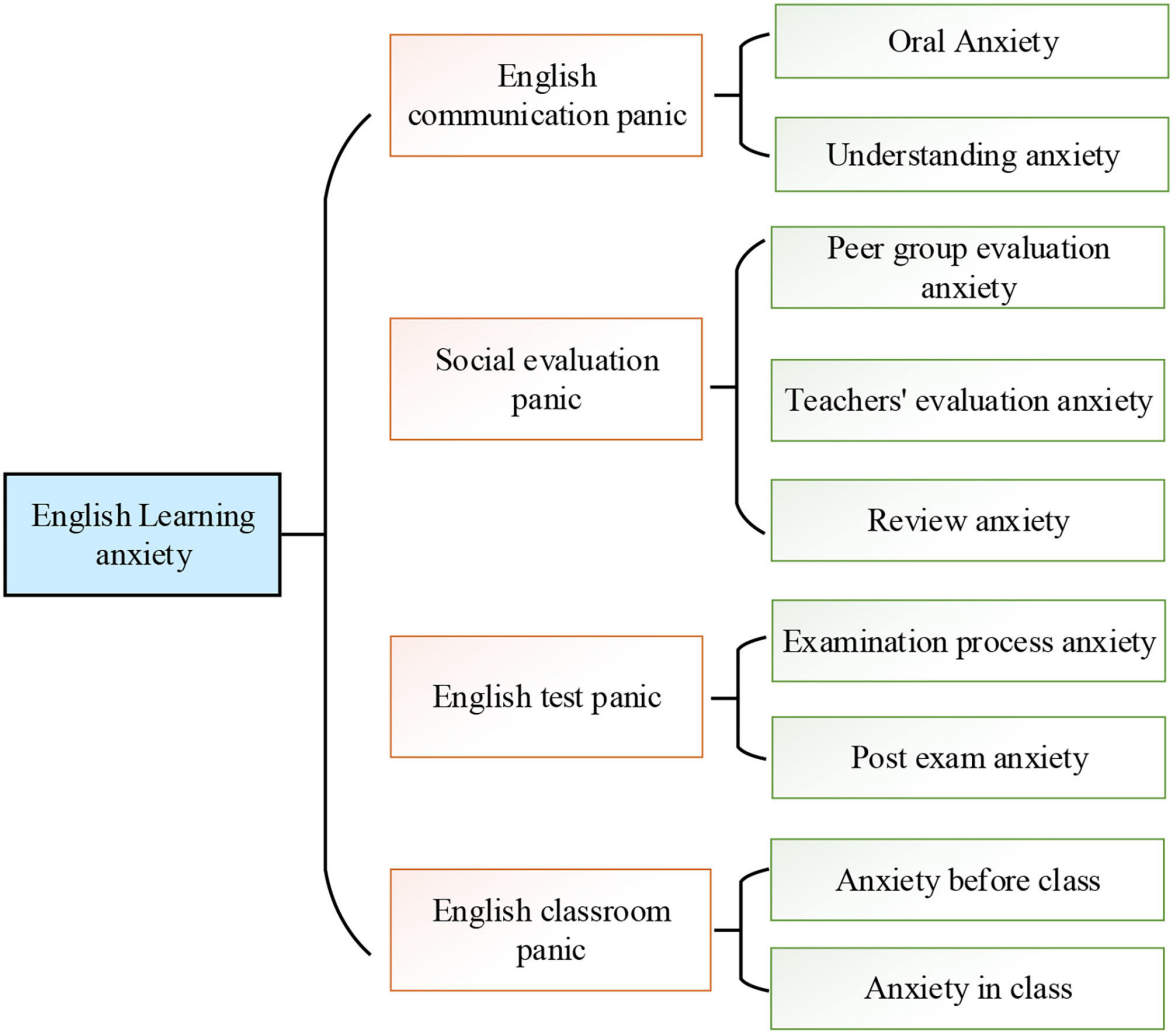
Students' English learning anxiety.

Generally, the psychological problems in English learning include negative emotions, such as tension, worry, and fear, which seriously affect the learning effects of the students (Gawi, 2020). English learning anxiety can be subdivided into three dimensions, namely, communication anxiety, negative evaluation anxiety, and test-taking anxiety. Communication anxiety refers to the fear or anxiety about real-life or expected communication with others (Du, 2020). Na (2018) pointed out that the typical behavior of communication anxiety was avoidance or withdrawal; compared with fearless speakers, these students were reluctant to get involved in the conversations or take part in the social activities. Negative evaluation anxiety refers to the fear and depression from negative evaluation, as well as the expectation for a negative evaluation of others. Test-taking anxiety refers to the fear of poor exam performances of the students. The English learning anxiety of the students is summarized, as shown in Figure 1. The anxiety of the students mainly comes from English communication, social evaluation, English test, and the English learning environment. In English teaching practice, the teachers sometimes raise questions without giving the students enough time for consideration, which is believed to increase the psychological burden of students, especially, the English learning anxiety.

### Vocalized Reading Practice Based on the Computer Simulation in Educational Communication System

A simulated intelligent learning platform based on AI technology can well-make up for the absence of a native linguistic environment in English learning, on which students can learn English anywhere and anytime by only using their mobile devices, and they can interact with their teachers or classmates freely. Meanwhile, the teachers can also use the intelligent learning platform to record the learning process of the students and analyze the evaluation results, thereby formulating better instruction courses for the students. The vocalized reading practice refers to the comprehensive application of phonetic knowledge and skills to transform static words into dynamic and emotional language expressions that can actively involve the multiple senses of an individual (Anwar, 2020). Some even regard the vocalized reading practice as an art form, while its roles are neglected by many in English learning. Thus, the roles of vocalized reading practice in language learning are summarized as follows.

The vocalized reading practice can help the students accumulate vocabulary, grammar, and cultural background, thereby broadening their horizons and improving their language expression proficiency. Further, the accumulated quantitative practices will eventually result in a spike in English performance, attended by uplifted self-confidence, and language skills (Gao et al., 2020). With the vocalized reading practice, the students can get an in-depth understanding of the reading materials, as well as an insight into the writing essence and literary style. In particular, it enables the students to feel the unique charm of English as a language (Calet et al., 2017). Through vocalized reading, the logical thinking and in-depth reading skills of the students can be significantly improved (Caldani et al., 2020).

The vocalized reading practice is the process of information processing by multiple body organs. An effective vocalized reading practice can form muscle pronunciation memory, deepen the brain's impression of word forms, and the understanding of the syntactic structure, and helps the students fabricate more sentences (Peng and Goodrich, 2020). The vocalized reading practice plays a critical role in the construction of sentence and discourse frameworks (Bourguignon et al., 2020). In English learning, the vocalized reading practice can cultivate the habit of oral expression of the students and help them build up solid memory for knowledge points, through which students can also feel the difference between phonemes and the changes in pronunciations (De Simone et al., 2021). Meanwhile, the vocalized reading practice can be used to train the pronunciation and intonation of the students, and it is also believed to be the best way to improve their listening and speaking skills. After long-term vocalized reading practice, the students can acquire a good sense of language and promote their written language automatically (Purwanto, 2019). Positive motivation, strong self-confidence, and low anxiety are believed to be favorable input information for the human language acquisition mechanism. On the contrary, lack of self-confidence, negative motivation, and strong anxiety are detrimental input information for the human language acquisition mechanism. In other words, creating a positive emotional experience using effective strategies is helpful in reducing the anxiety of students in practicing oral English. The teachers can alleviate the language learning anxiety of students by arousing their English learning interest and creating a comfortable English learning atmosphere.

Situational learning theory holds that learning is a social, practical, and differential resource-mediated participation process. It emphasizes that the students need to learn specific knowledge in a situational learning environment, that is, English learners need to learn English in a specific linguistic environment, through dialog and other interactive exercises in real or simulated real situations to achieve better learning results. The computer simulation in education communication (CSIEC) is an intelligent English learning system and includes an AI robot for chatting, a teaching platform, and software to recite English words (Choi et al., 2020). The chat robot can create a specific situation in which a dialog is proceeding naturally, and simulate the environment of the dialog in the text. The teaching platform mainly aims at the classroom learning needs of students, provides learning materials for them, and gives scores and feedbacks timely every time the students complete the exercises.

### Self-Efficacy Theory in English Learning

According to Bandura's theory, the beliefs of a learner (self-efficacy) refer to the faith of a person in self-competence to complete specific tasks, which can help students complete a certain task in the learning process. Many studies on educational psychology have proved the role of self-efficacy in language learning (Namaziandost and Çakmak, 2020). Moreover, self-efficacy serves as a medium to transform knowledge into action and emphasizes the importance of the beliefs of the learners and motivation in learning. Therefore, to effectively implement knowledge and skills into practice, the self-efficacy of students should be cultivated and enhanced (McBride et al., 2020).

Self-efficacy regulates human function through cognition, drive, emotion, and decisions. All of these affect the self-recognition of an individual, that is, when individuals encounter difficulties, the self-efficacy will encourage them to persist and deal with the pressure rationally and firmly (Perera et al., 2019). Syaful Anam and Elke Stracke studied the relationship between the learning strategies selection and self-efficacy of Indonesian students in English learning in a primary school (Margahana, 2019); the results showed that students with the higher self-efficacy adopted different learning strategies from those with lower self-efficacy because they believed in themselves in undertaking English learning tasks and could regulate their learning behaviors. This shows that self-efficacy influences the learning effects of students (Yao et al., 2019).

Currently, the relationship between the learning effect and self-efficacy has been proved by many scholars. However, the research on the role of vocalized reading practice in enhancing self-efficacy is very limited (Dorfman and Fortus, 2019).

It has been argued that self-efficacy is not inborn but forms through daily activities, such as learning and practice. Reasonable teaching methods play an important role in improving self-efficacy (Burnette et al., 2020). It is necessary to arouse the interest of the students in English learning and improve their scores through the improvement of the self-efficacy of students, thus realizing their virtuous development (Marsh et al., 2019).

### Topic Familiarity in English Learning

Cognitive science states that the learners' understanding of the topic depends on two factors. One is knowledge reserve, such as the general knowledge structure, also known as background knowledge or schema; the other is the information of the topic, namely, the meaning of the topic (Qiu, 2020). The cognitive linguists contend that background knowledge can be used for understanding and accepting new knowledge, which is beneficial to English language learning. If the topics fall into the schemata of learners, they will show cognition or awareness, have a clear understanding of the topic, and express themselves fluently and logically around the topic (Yang and Kim, 2020). In particular, when the topic arouses the interests of students, the speaker might speak more based on their own experiences (Klassen, 2020). In schema theory, an oral expression is considered an agile thinking activity. The students can interact with the materials and their schemata through the topic, and activate, reconstruct, and reproduce their expression in different ways to articulate logical sentences. Therefore, the activation of the corresponding schema in the brain may greatly affect the learning content of students (Intini et al., 2019).

The topic is a key factor of oral performance both in the oral tests and classroom activities. The different oral topics will show distinct difficulties for the students. Therefore, it is necessary to combine the topic familiarity with oral English performance. Thus, the topic selection should be based on the prior experiences of students while bringing new experiences. Most students usually fall into a loose state and seldom reflect on theirs lives. Thus, the teachers might think of better topics to stimulate the students to communicate with each other more, share their life experiences, express their thoughts in the classroom, thereby improving the topic awareness of students in English learning.

## Research Methods and Models

### Research Hypothesis

The specific implementation process of the vocalized reading practice is as follows. The same English teacher from the control class will implement the vocalized reading practice in the experimental class by arranging self-study course and pronunciation guidance course, as well as various forms of reading practices. The present study aimed to help the students master reading skills, stimulate their interest in reading and learning, and help them overcome the psychological problems in learning English. The vocalized reading is a basic skill that the students are supposed to master, especially, when learning English, which can significantly improve their oral English; besides, it is the basis of memorization and an efficient way to learn English pronunciation and intonation; hence, the vocalized reading practice plays a positive role in stimulating the interest of students in learning and improving the quality of English teaching; additionally, the vocalized reading practice can cultivate the enthusiasm of students in learning English by changing negative learning attitudes toward the positive learning attitudes, and it can cultivate the enterprising spirit of students for active research and autonomous learning.

Taboada Barber et al. (2015) analyzed the role of vocalized reading practice in improving the reading comprehension ability and reading self-efficacy of middle school students under different language backgrounds. The group intervention and test suggested that the vocalized reading practice positively impacted the English comprehension and English learning self-efficacy of students. Accordingly, this study puts forward the hypotheses H1, H3, and H5. Hong et al. (2014) discussed the anxiety caused by the low self-efficacy of students in English learning, finding that the long-term low English scores would lead to the decline of self-efficacy, resulting in English learning anxiety of the students. Based on this, the present study puts forward the hypotheses H2, H4, and H6.

Before the implementation of the research method, the following hypotheses are put forward.

H1: The vocalized reading practice can improve the topic familiarity of students in English learning.
H2: The higher the topic familiarity of students in English learning, the lower is their English learning anxiety.
H3: The vocalized reading practice can improve the self-efficacy of students in learning English.
H4: The higher the self-efficacy of students in English learning is, the lower the degree of English learning anxiety is.
H5: The vocalized reading practice can improve the English scores of students.
H6: The higher the English scores of students are, the lower is their English learning anxiety.

### Experimental Design and Research Methods

The purpose of the experiment is to explore the influence of vocalized reading practice on the topic of familiarity and self-efficacy of students in English learning, as well as English learning anxiety and the psychological problems of students in language learning. An experimental comparison method was adopted to analyze the effect of vocalized reading practice on the self-efficacy and English grades of students. The two classes of students with close English scores in grade two of S middle school in Suzhou, Jiangsu Province, China were recruited as respondents. S middle school ranks among the medium in local educational institutions in terms of teaching quality, and the English level of the respondents is moderate among the overall grade two students, which ensures the representativeness and authenticity of the research results. In total, 86 students in the two classes were involved in the experimental research, of which 44 students in class 1 were assigned to the experimental class, and 42 students in class 2 were assigned to the control class. The proportion of the boys and girls in the two classes was evened up.

In this study, several methods were used, such as control experiment, interview, and a QS. The experimental teaching method was used to explore the effectiveness of the vocalized reading practice on improving the oral English proficiency of middle school students. Meanwhile, the more flexible semi-structured interview rather than the standardized interview was used for the middle school students given their particular age. The interviewer and the respondents were allowed to communicate freely during the interview according to the outline.

The control experiment was conducted on the experimental class and the control class for one semester. Specifically, the experimental class adopted the proposed reading strategies: to read according to guidance, read with pause, and read with speed. The control class was guided by the general reading materials. The control experiment chose the same teaching content for the two classes whose teaching progress were consistent, and in-class practice and after-class practices were all designed with the same contents; the experimental class was given an extra 20-min of vocalized reading practice compared with the control class which just received the routine teaching.

Then, the QS method was used. The topic familiarity scale was used to conduct the pre-test and post-test on the topic familiarity of the students in the two classes. The QS was divided into three dimensions: familiarity of cultural background knowledge, the familiarity of life experience, and familiarity of contextual meaning, which were used to investigate the students' mastery of social and cultural backgrounds, social experiences, the relationship between the context and language expression, and the explicit and implicit meanings of the reading materials. The QS contained 20 topics and was scored with a Likert five-level scale, which was consistent with the research questions. The higher the score is, the more familiar the students are with the topic in English learning. The English Learning Self-Efficacy Scale was used to conduct pre-test and post-test on the self-efficacy of two classes; the scale subdivided the self-efficacy into two parts: learning ability and learning behavior; learning behavior can reflect the motivation of students to complete learning; there were 10 questions in each dimension, with a total of 20 questions; five-point evaluation system was used to obtain the score of the QS; the higher the score is, the higher the sense of self-efficacy is; the English Learning Self-Efficacy Scale was used for the self-efficacy pre-test and post-test in the experimental class. Afterward, the Foreign Language Classroom Anxiety Scale (FLCAS) was used to measure the English learning anxiety and psychological problems of the students; the scale consists of 20 items, and four dimensions: communication anxiety, test-taking anxiety, negative evaluation anxiety, and general anxiety. The Likert five-point scoring method was used to calculate the score of the QS. The higher the score is, the more serious the anxiety of the student is.

The purpose is to study the influence of vocalized reading practice on the topic familiarity and self-efficacy of the students, as well as English grades improvement and overcoming their psychological problems in English learning. The teacher-tested and scored oral English of students before and after the vocalized reading practice.

The students filled out the QS anonymously, and their personal information was kept confidential. The vocalized reading practice lasted about 15–20 min for every section. The QS was distributed and recovered on the spot. The recovered effective QSs were statistically analyzed using SPSS 21.0, and *P* < 0.05 showed that the collected data had statistical significance. The results showed that the self-efficacy of students in the experimental class and control class was significantly different.

## Results and Discussion

### Descriptive Analysis Results of Key Variables

**Figure 2:**
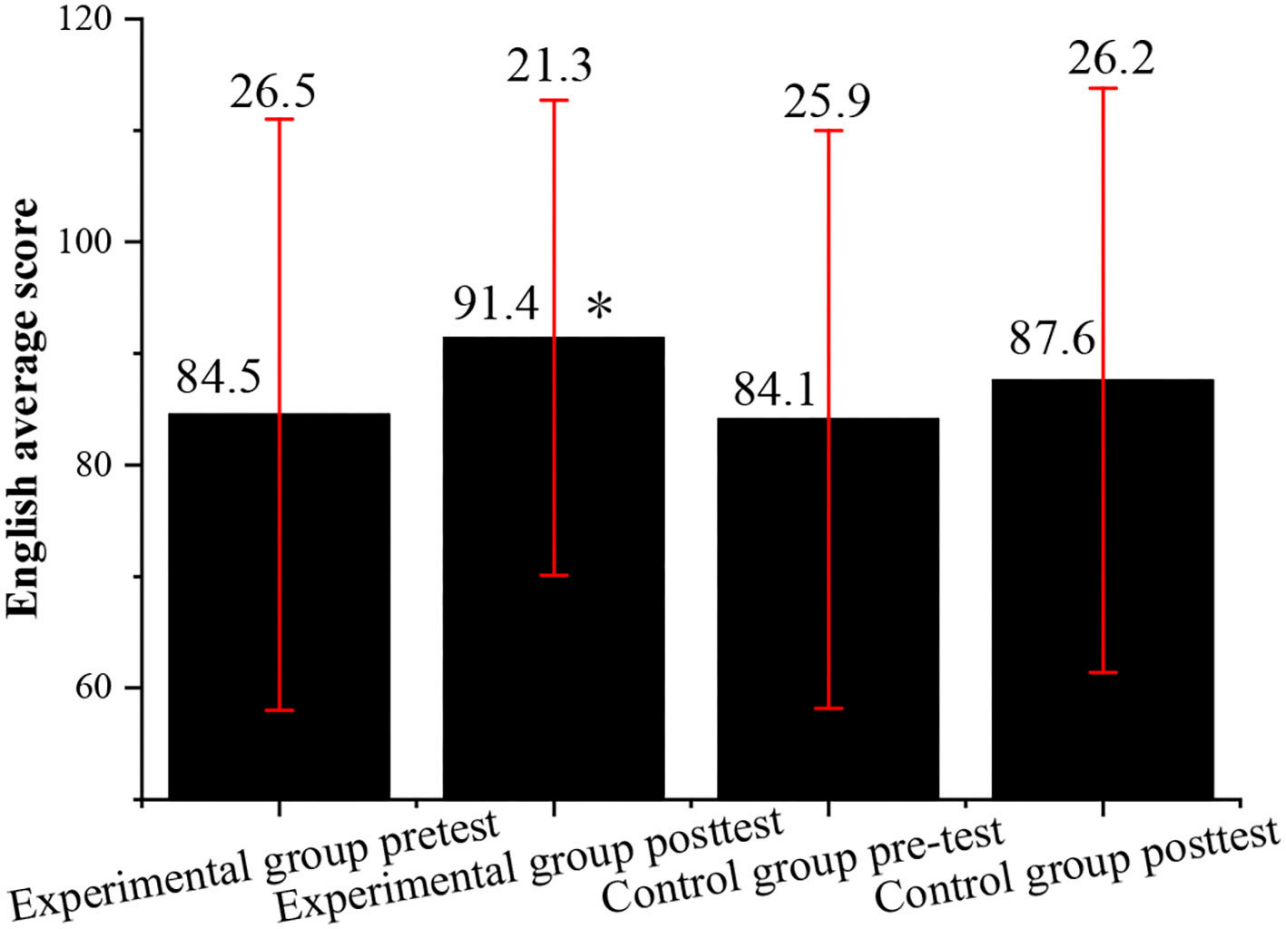
Comparison of the average English scores between the experimental class and the control class before and after the experiment (*indicates *P* < 0.05 compared with the post-test of the control class).

Before the experiment, the English scores of the experimental class and the control class are tested and compared. After the experiment, the paired-sample *t*-test and an independent sample *t*-test are conducted on the English scores of the two classes. Figure 2 shows the statistical results.

After the experiment, the average score of the experimental class is greatly improved, but the SD is reduced, which shows that the English scores of the students are more evenly distributed than before, and the learning effect is improved significantly. The average score of the control class increases, but the SD continues to rise, which indicates that there is still a big gap among the English scores of the students in the control class after the experiment, which has shown no significant improvement from pre-test English scores. The comparison of the average English scores reveals that the scores of the two classes show an upward trend; but the score improvement in the control class is not significant, while that of the experimental class is higher than the control class. Moreover, the reduction of SD indicates that the score gap in the experimental class is narrowed, which proves that the vocalized reading practice has a certain effect on improving the overall English grades of the students.

Subsequently, the effectiveness of the experimental results is further studied through post-test and pre-test. The students in the control class and the experimental class are required to complete the QS of English learning self-efficacy at the same time. The results are analyzed as follows.

**Figure 3:**
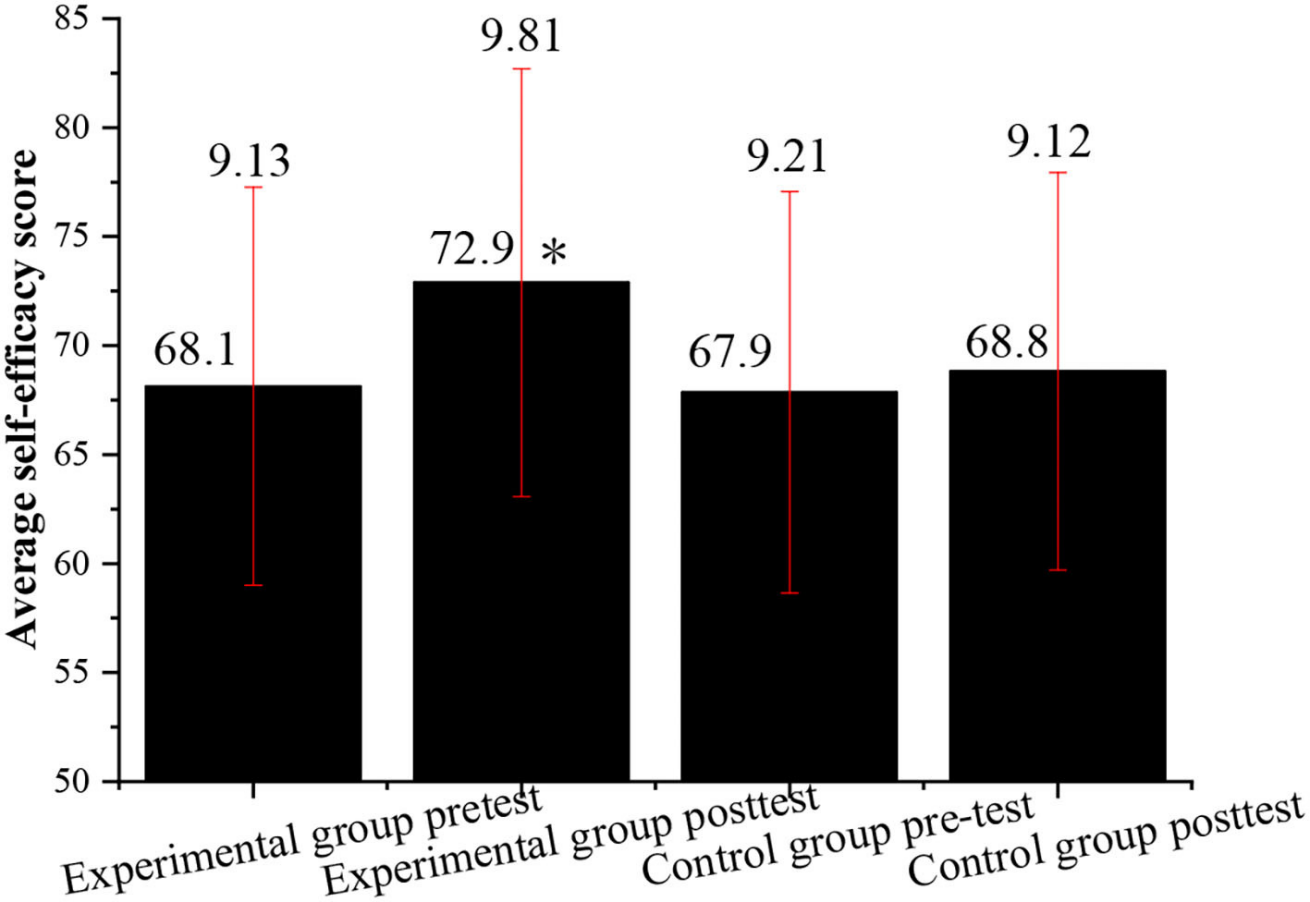
Comparison of the average score of English learning self-efficacy between the experimental class and the control class before and after the experiment (*indicates *P* < 0.05, compared with the post-test of the control group).

Figure 3 shows that before the vocalized reading practice experiment, the difference in the average score of English learning self-efficacy between the experimental class and the control class is very small, that is, the level of self-efficacy of the two classes is roughly the same. However, after the experiment, the scores of English learning self-efficacy of the experimental class are significantly improved, while the scores of the control class are almost unchanged. This shows that the English learning self-efficacy of the students in the experimental class is greatly enhanced, and the English learning self-efficacy of students in the control class is basically the same before and after the experiment.

**Figure 4:**
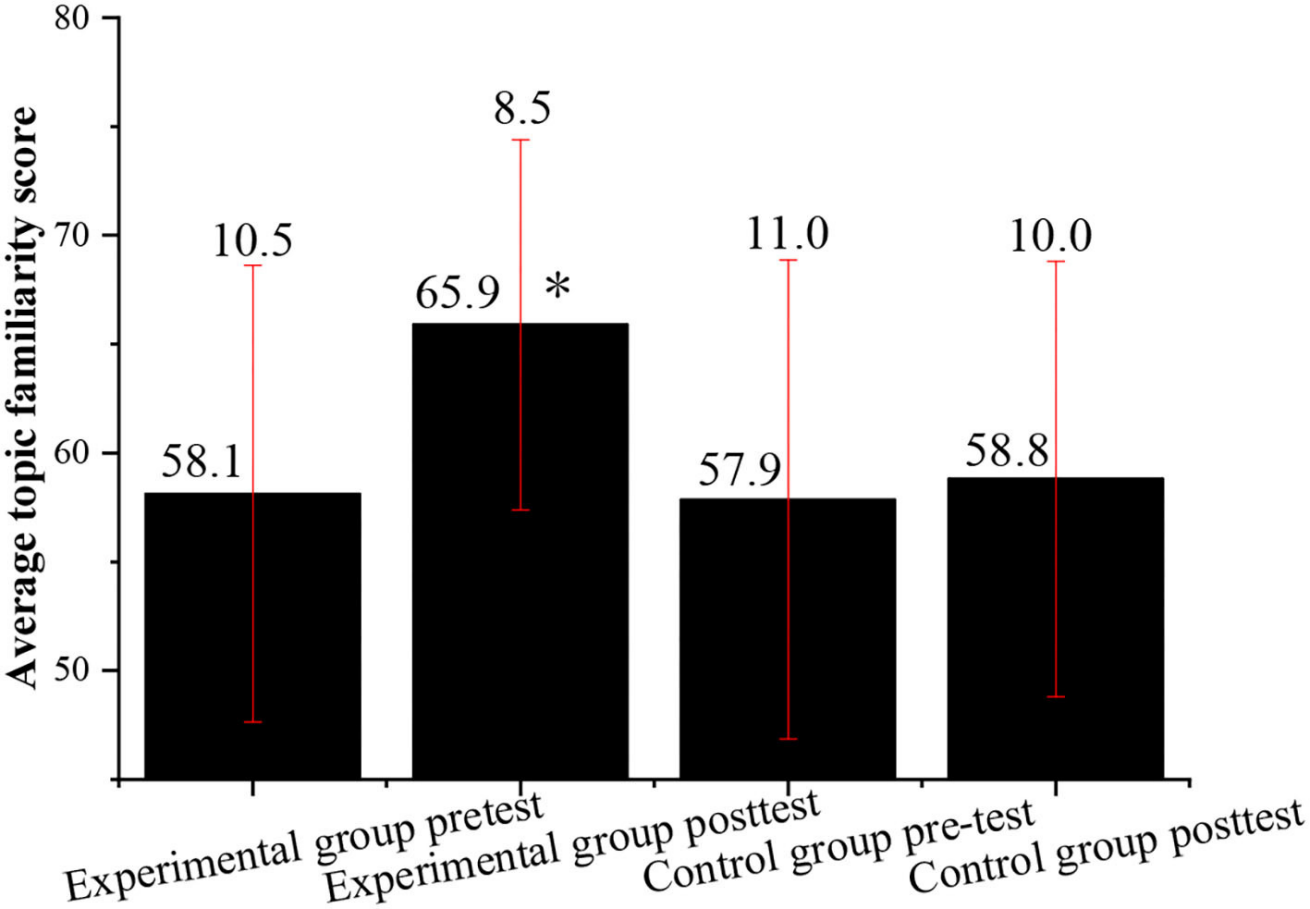
Comparison of the average score of topic familiarity in English learning between the experimental class and the control class before and after the experiment (*indicates *P* < 0.05 compared with the post-test of the control group).

Next, the topic familiarity of the experimental class and that of the control class are investigated through post-test and pre-test. The students in the control class and the experimental class are required to complete the English learning topic familiarity scale at the same time to better prove the effectiveness of the experimental results. Figure 4 shows the QS results.

Figure 4 shows that before the experiment, the difference in the average score of the topic familiarity in English learning between the experimental class and the control class is very small, that is, the topic familiarity of the two classes is roughly the same. However, after the experiment, the scores of the topic familiarity of the experimental class are significantly improved, while the scores of the control class are almost unchanged. This shows that the students in the experimental class with the vocalized reading practice have a better mastery of the topic in English learning, while the students in the control class without the vocalized reading practice have almost the same topic familiarity in English learning before and after the experiment.

**Figure 5:**
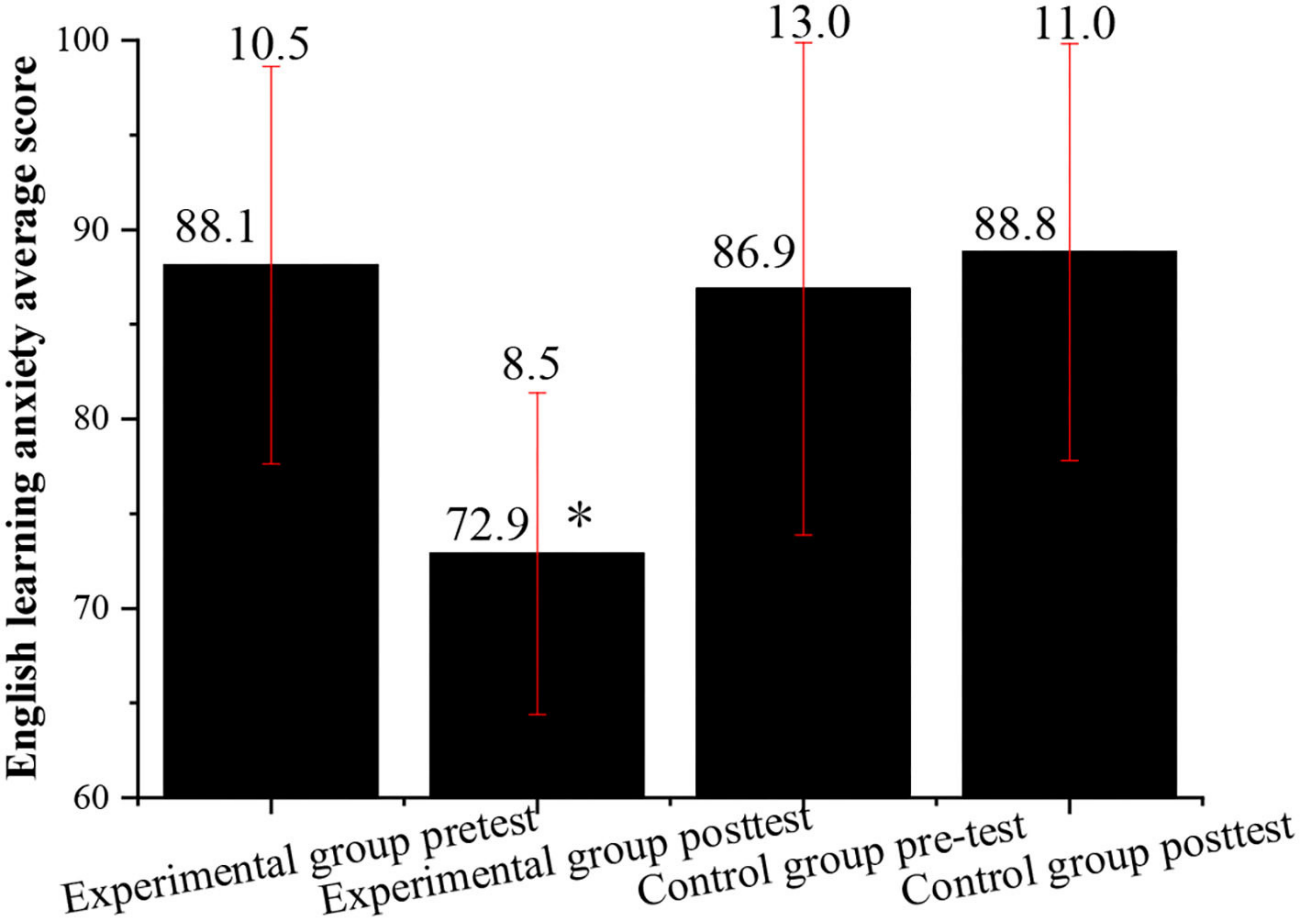
Comparison of the average score of English learning anxiety between the experimental class and the control class before and after the experiment (*indicates *P* < 0.05 compared with the post-test of the control group).

Then, the English learning anxiety is investigated for the experimental class and the control class through pre-test and post-test. The students in the control class and the experimental class are asked to complete the English learning anxiety scale at the same time to ensure the effectiveness of the experimental results. Figure 5 shows the QS results.

Figure 5 shows that before the experiment, there is a small difference in the average score of English learning anxiety between the experimental class and the control class, that is, the degree of English learning anxiety of the students in the two classes is roughly the same. However, after the experiment, the scores of the English learning anxiety of the students in the experimental class decrease significantly, while the scores of the control class hardly change or even increase. This suggests that the English learning anxiety of the students in experimental class with the vocalized reading practice is greatly reduced, while the English learning anxiety of the students in control class is the same before and after the experiment.

Figures 2–5 indicate that the vocalized reading practice is simple and easy to be used, and it has various forms and can arouse the learning enthusiasm of the students, specifically, for those students with learning difficulties. Through the vocalized reading practice, the students can read English texts skillfully and fluently, understand the meaning of the text, and learn cultural backgrounds around English. Meanwhile, while reading aloud students can also stimulate their multiple sensory organs by listening to themselves, which can help to improve their listening skills by practicing pronunciation and getting themselves familiar with the characteristics of oral English.

### Analysis of the Correlation Between the Topic Familiarity of the Students in Learning English and Their English Learning Anxiety

The post-test topic familiarity of the students and their English learning anxiety are plotted to show the influence of topic familiarity on the English learning anxiety of the students under the vocalized reading practice.

**Figure 6:**
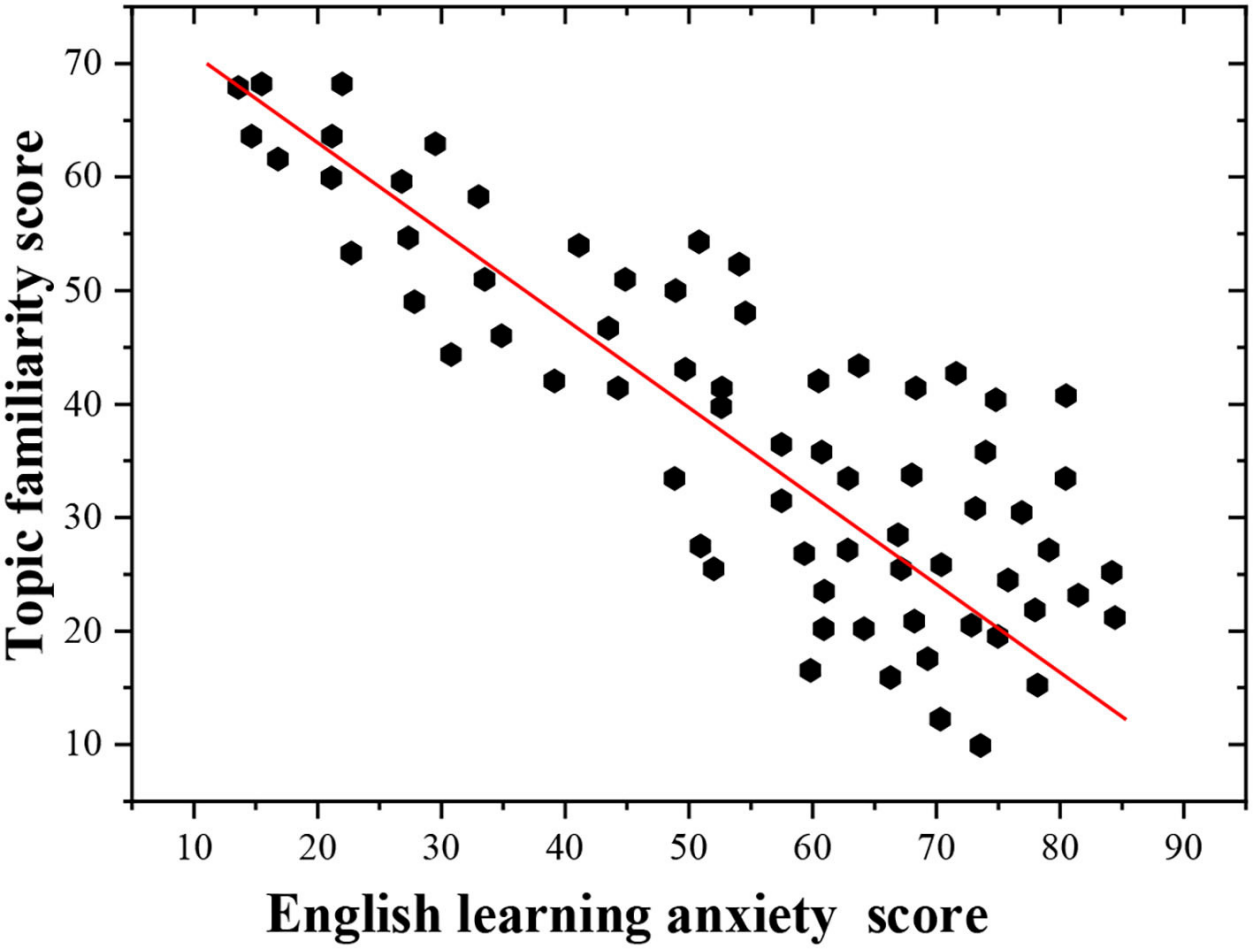
Scatterplot of topic familiarity and English learning anxiety.

As Figure 6 suggests, the scatter between the topic familiarity and the English learning anxiety shows a downward straight line from the upper left corner to the lower right corner, which indicates that there is a negative linear relationship between the topic familiarity and English learning anxiety. The more familiar the students are with the topic, the lower the English learning anxiety is.

The regression analysis is conducted to fully understand the relationship between the topic familiarity and English learning anxiety. The topic familiarity is taken as an independent variable, while English learning anxiety is used as the dependent variable. The regression analysis (Table 1) shows that the topic familiarity can be introduced into the regression model, indicating that the topic familiarity can partly predict the English learning anxiety of the students and is closely related to their English learning anxiety. The higher the students' mastery of the topic content is, the lower the degree of English learning anxiety is. Thus, the hypotheses H1 and H2 hold.

**Table 1:** Regression coefficient of the topic familiarity and English learning anxiety.

	**Unstandardized coefficients**		**Standardized coefficients**	***t***	**Significance**
	***B***	**Standard error**	**Beta**		
Constant	165.685	2.563		243.457	0.000
Topic familiarity	−2.312	0.000	−0.967	−92.634	0.000

*Dependent variable: English learning anxiety*.

The regression results show that topic familiarity has a good predictive effect on the English learning anxiety, and the model significance is *P* = 0.000 <0.05, indicating that the whole regression model has statistical significance. In the regression coefficient table, the *t*-value is 243.457, and the coefficient significance of the model is *P* = 0.000 <0.05, indicating that the original hypothesis data can be accepted, and the whole model is valid.

Hence, the final regression equation is deduced: English learning anxiety = 165.685 – 2.312 ^*^ topic familiarity.

### Analysis of the Correlation Between the Self-Efficacy in English Learning and English Learning Anxiety

The scatterplot of post-test self-efficacy in English learning and English learning anxiety of respondents is drawn to show the effect of self-efficacy in English learning anxiety of the students under the vocalized reading practice.

**Figure 7:**
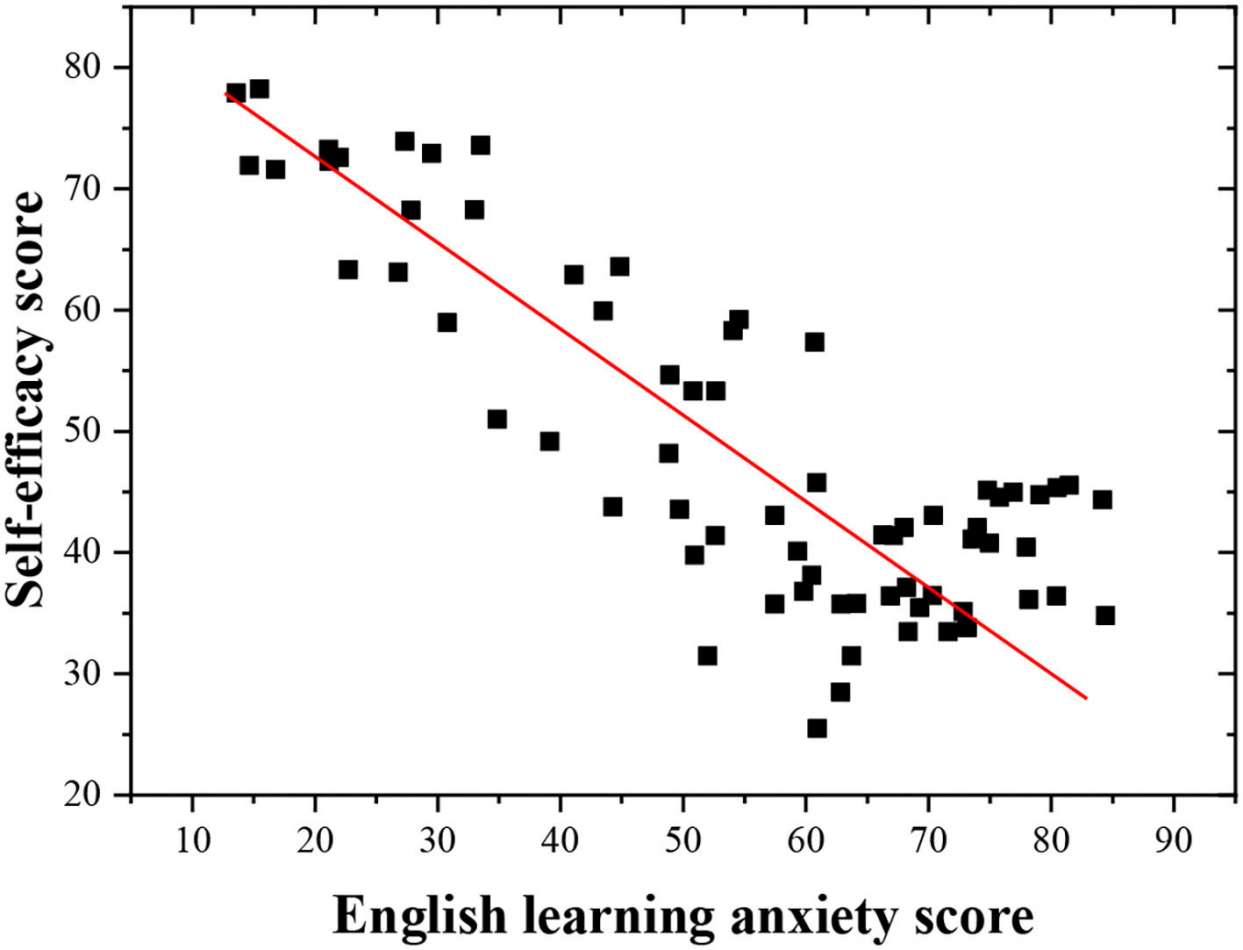
Scatterplot of the self-efficacy and English learning anxiety.

The scatter between self-efficacy and English learning anxiety shows a downward trend from the upper left corner to the lower right corner, indicating that there is a negative linear relationship between the self-efficacy of the students in English learning and English learning anxiety (Figure 7). According to the scatterplot, the higher the self-efficacy of students is, the lower their English learning anxiety is. Self-efficacy is taken as an independent variable, and English learning anxiety is taken as the dependent variable for the regression analysis to fully understand the relationship between self-efficacy and English learning anxiety of middle school students. The regression analysis (Table 2) suggests that self-efficacy can be introduced into the regression model, indicating that self-efficacy can partly predict the English learning anxiety of the students, thus proving that self-efficacy is closely related to the English learning anxiety of the students. Thus, the hypotheses H3 and H4 hold.

**Table 2:** Regression coefficient of the self-efficacy and English learning anxiety of the students.

	**Unstandardized coefficients**		**Standardized coefficients**	***t***	**Significance**
	***B***	**Standard error**	**Beta**		
Constant	143.685	3.257		178.586	0.000
Self-efficacy	−1.834	0.000	−0.893	−85.439	0.000

*Dependent variable: English learning anxiety*.

The regression results show that the self-efficacy has a good predictive effect on the English learning anxiety, and the model significance *P* = 0.000 < 0.05, indicating that the whole regression model has statistical significance. In the regression coefficient table, the *t*-value is 178.586, and the coefficient significance of the model is *P* = 0.000 < 0.05, indicating that the original hypothesis data can be accepted, and the model is valid. Hence, the final regression equation is deduced: English learning anxiety = 143.685 – 1.834 ^*^ self-efficacy.

In summary, the students should be cultivated with empathetic reading and learning skills based on their full understanding of the reading materials and imaginations, which can be perfectly realized through the vocalized reading practice. Meanwhile, the teachers should take individual differences into account while striving to devise a universal learning method for the students in the vocalized reading practice. The students with outstanding reading performances should be honored and encouraged, thereby cultivating their sense of accomplishment, which in turn, can strengthen their aspiration for the vocalized reading, thus forming a higher sense of self-efficacy.

### Analysis of the Correlation Between English Grades and English Learning Anxiety

**Figure 8:**
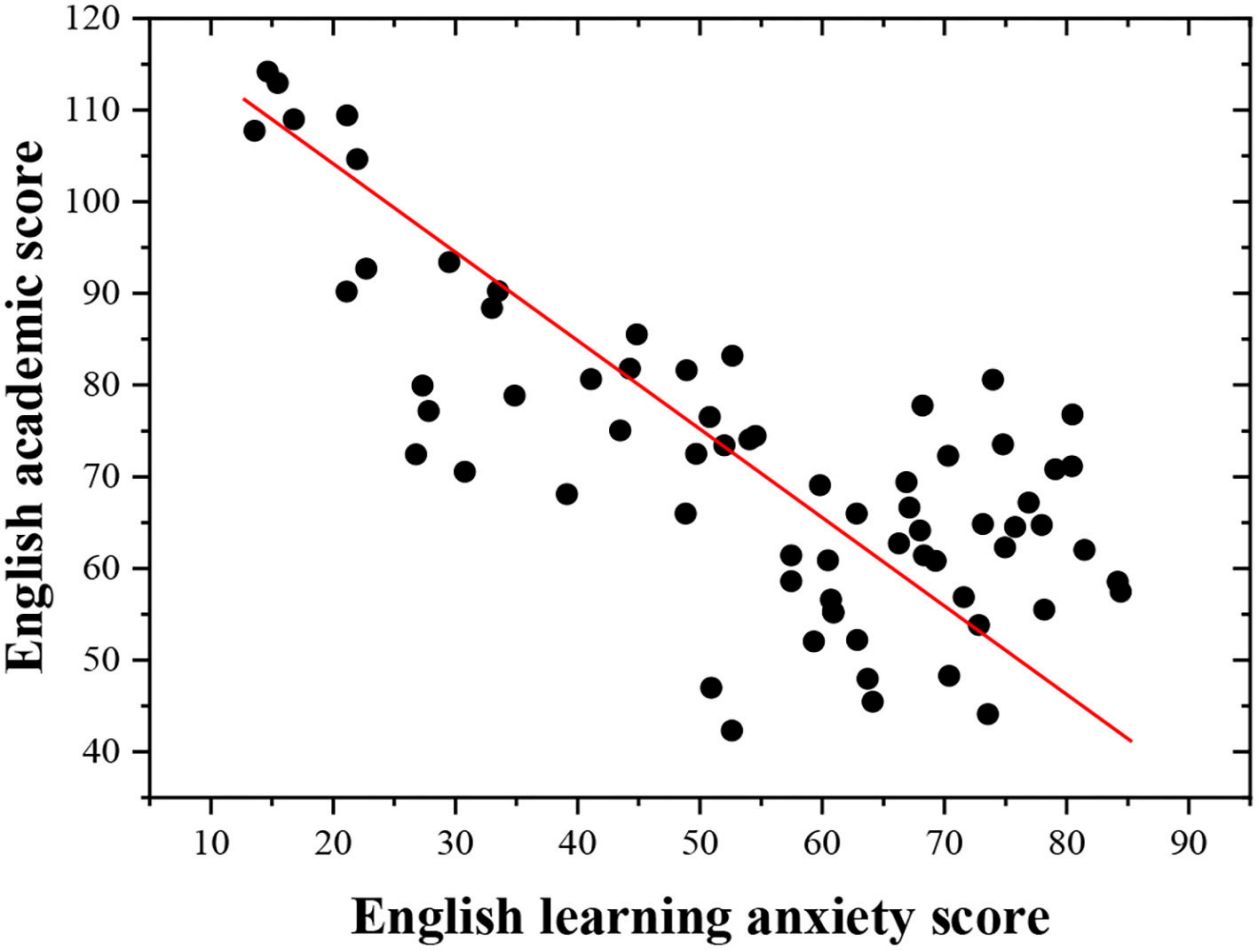
Scatterplot of the English grades and English learning anxiety of students.

The scatterplot of post-test English learning scores and English learning anxiety of respondents is drawn to show the influence of English grades of the students on their English learning anxiety under the vocalized reading practice (Figure 8).

The scatter between the English grades and English learning anxiety of students shows a downward trend from the upper left corner to the lower right corner, indicating that there is a negative linear relationship between the English grades and English learning anxiety of students. The better their English grades are, the lower their degree of English learning anxiety is.

The regression analysis is conducted on the English grades and English learning anxiety of the students to fully understand the relationship between the English grades and English learning anxiety. The English grades of the students are taken as the independent variable, and learning anxiety is taken as the dependent variable. The regression analysis (Table 3) reveals that the English grades of the students can be introduced into the regression model, indicating that the English scores can partly predict their English learning anxiety and English grades of the students are closely related to their learning anxiety. The better the English grades of the students are, the lower the degree of English learning anxiety is. Thus, the hypotheses H5 and H6 hold.

**Table 3:** Regression coefficient of English grades and English learning anxiety of the students.

	**Unstandardized coefficients**		**Standardized coefficients**	***t***	**Significance**
	***B***	**Standard error**	**Beta**		
Constant	178.685	2.875		185.742	0.000
English scores	−1.268	0.000	−0.846	−75.469	0.000

*Dependent variable: English learning anxiety*.

The results show that English grade has a good predictive effect on English learning anxiety, and the model significance is *P* = 0.000 < 0.05, indicating that the whole regression model has a statistical significance. In the regression coefficient table, the *t*-value is 185.742, and the coefficient significance in the model is *P* = 0.000 < 0.05, showing that the original hypothesis data can be accepted, and the model is valid. Hence, the final regression equation is deduced: English learning anxiety = 178.685 – 1.268 ^*^ English grades of the students.

## Discussion

The vocalized reading practice is an essential method in English learning, which is simple and interesting, during which, the teachers should create a relaxed and pleasant reading environment for the students, reward them with excellent performance, and cultivate them with a correct view on success or failure, as well as difficulties and achievements. Meanwhile, there is a need for the teachers to let students positively evaluate their self-learning ability, understand the essence of learning, discard negative outlooks on learning, and arduously face difficulties in learning. The vocalized reading practice can help the students accumulate English vocabulary, develop English thinking habits, and cultivate English language sense and imagination. On this basis, the students will be relaxed and be more fluent at expressing themselves in writing. By developing their listening, speaking, reading, and writing skills in English, the students can get a sense of accomplishment in learning. Meanwhile, the English performance of the students will be improved, which, in turn, helps to uplift their confidence and self-efficacy in learning English.

This study conducted a controlled experiment and a QS, and the data results are statistically analyzed and tested using the regression model. The results show that the average scores of self-efficacy, English scores, and topic familiarity of the students in the experimental class are significantly improved, while the average scores of the students in the control class almost have not changed. This shows that the vocalized reading practice has a certain effect on improving the English scores, self-efficacy, and topic familiarity of the students. The results of the regression analysis prove that the English scores, self-efficacy, and topic familiarity can better predict English learning anxiety. Overall, there is a significant correlation between English learning anxiety and English learning self-efficacy, topic familiarity, and English scores. Ardasheva et al. (2018) pointed out that the correlation between learning anxiety and self-efficacy was confirmed through the data analysis, which was proved in this study. Zhang et al. (2020) also confirmed that the positive psychological state of the students could promote the smooth development of teaching activities and strengthen the teaching effect.

It has been contended that if the learners experience too much frustration and anxiety while learning, their initially low self-efficacy may completely change to learned helplessness, and, as a result, they may give up continuing efforts and attempts. It is found that in the process of teaching, some students are completely uninterested in learning. They are indifferent toward the success or failure in learning, despite the unremitted efforts of the parents or teachers. It helps the students to learn to notice their negative anxiety consciousness and experience in time and talk about them with their teachers. Moreover, the teachers should cultivate the students with a challenging spirit against learning anxiety by strengthening their positive behaviors and encouraging their slightest progress.

To help the students shape effective English learning strategies, the English teachers should emphasize the importance of vocalized reading practice. In particular, the students should be trained with social strategies to create a learning environment conducive to overcoming the psychological anxiety of the students, thus improving their learning effect.

## Conclusion

English learning, like other language learnings, is a long and arduous process, in which a suitable linguistic environment is essential for reading and oral practices. The application of the CSIEC system based on AI in English classroom teaching can create an English linguistic environment for the students, provide functions, such as words dictations, listening exercises, and timely feedback. As an intelligent English learning platform, the CSIEC can also stimulate the interest of learners in learning and improve their learning motivation.

Here, the vocalized reading practice in English learning is specifically discussed to study its influence on the English learning anxiety of the students based on educational psychology, the theory of self-efficacy, and the schema theory. Some hypotheses are put forward, concerning the effects of self-efficacy, topic familiarity, and English grades on the English learning anxiety of the students under the vocalized reading practice. The results suggest that the vocalized reading practice can improve the topic familiarity of the students in English learning and their English scores, thus reducing the English learning anxiety and improving their self-efficacy in English learning.

This study adopts a specific and operable educational intervention to help the students with difficulties in English learning. Additionally, this study has great significance to promote each student to obtain maximum development. Due to the limited conditions, the tests on the respondents cannot be conducted. The selected respondents are from the two classes in one school, which cannot represent all the students, and the experimental results are not universal.

## Data Availability Statement

The original contributions presented in the study are included in the article/Supplementary Material, further inquiries can be directed to the corresponding author/s.

## Ethics Statement

The studies involving human participants were reviewed and approved by Zhoukou Normal University Ethics Committee. The patients/participants provided their written informed consent to participate in this study. Written informed consent was obtained from the individual(s) for the publication of any potentially identifiable images or data included in this article.

## Author Contributions

All authors listed have made a substantial, direct and intellectual contribution to the work, and approved it for publication.

## Conflict of Interest

The authors declare that the research was conducted in the absence of any commercial or financial relationships that could be construed as a potential conflict of interest.

## Publisher's Note

All claims expressed in this article are solely those of the authors and do not necessarily represent those of their affiliated organizations, or those of the publisher, the editors and the reviewers. Any product that may be evaluated in this article, or claim that may be made by its manufacturer, is not guaranteed or endorsed by the publisher.
