# Supplementary material for: Influence of Vocalized Reading Practice on English Learning and Psychological Problems of Middle School Students
Source: Front Psychol. 2021 Oct 18;12:709023. doi: 10.3389/fpsyg.2021.709023 (PMC8558255; doi:10.3389/fpsyg.2021.709023)
Supplement: Supplementary file 1 [file Data_Sheet_1.pdf]

Appendix

QS on the self-efficacy of students' English learning

There are five options for each question, which are completely inconsistent, inconsistent, basically inconsistent, uncertain, basically consistent, and completely consistent.

1. I can master the content taught by the English teacher in time in class.
2. If I want to learn some new English knowledge now, I believe I can learn it well.
3. In English class, I often can't concentrate on listening.
4. If I have difficulty in learning a certain English language knowledge or grammar rules, I believe I can learn it quickly.
5. I can easily deal with all kinds of English tests.
6. I often don't know how to make an English study plan according to my actual situation.
7. I can solve all kinds of difficulties in English learning.
8. If the English teacher let us read the textbook by ourselves, I often don't know what to read.
9. If I am asked to solve a difficult English problem, I will feel incompetent.
10. I can always finish my English homework well.
11. I think English is becoming more and more difficult to learn, and I can't learn it well.
12. I believe I can always find effective learning methods to learn English well.
13. If the teacher asks us to solve a practical problem related to English, I can find a suitable way to solve it.
14. I am often very nervous when the teacher asks questions in English class.
15. If the English teacher asks me to explain a word or sentence in English, I believe I can explain it clearly.
16. I like to choose some challenging English learning tasks.
17. I can use my English knowledge and grammar rules to solve related English problems.
18. If the teacher asked me to do an English exercise, I believe I can do it.
19. Even if my score on an English test is not ideal, I can calmly analyze the reasons and continue to work hard.
20. I don't think I'm good at learning English.

QS on English Learning

Please tick "√" in the option that suits your situation.

1. Are you interested in learning English now?

1. Very interested 2. Quite interested 3. A little interested 4. Not interested

2. Your English scores belong to

1. Good 2. Middle to good 3. Middle 4. Middle to poor 5. Poor

3. Apart from English class, how much time do you spend on English study every day?

1. More than 1 hour 2. About 1 hour 3. Half an hour 4. No

4. Will you actively exchange English learning experiences and methods with others?

1. Often 2. Sometimes 3. Rarely 4. Never

5. Do you have the habit of previewing the text?

1. Often 2. Sometimes 3. No

6. How do you finish your homework?

1. Finish the homework independently 2. Discuss with classmates 3. Copy others' homework 4. Don't do it

7. If you make mistakes in the homework, will you do it again every time?

1. Definitely. 2. Most of the time. 3. Occasionally. 4. Never

8. What do you usually do when you encounter difficulties in English learning?

1. Ask for advice from classmates 2. Ask for help from teachers 3. Try my best to find a way 4. Give up

9. What do you often do when the teacher asks questions in class?

1. Raise my hand when I am not sure of the correct answer 2. Do not raise my hand when I know the correct answer 3. Do not raise my hand when I am not sure of the correct answer 4. Wait for other students to raise their hands to answer

10. What do you usually do when you read unfamiliar words?

1. Guess the meaning of new words by context 2. Solve new words by looking up dictionaries 3. Skip new words 4. Others______

11. Your mastery of English grammar

1. Be familiar with grammar rules and be able to use them flexibly. 2. General mastery. 3. Poor

12. How do you learn English grammar?

1. Strengthen grammar rules by doing lots of grammar tests 2. Do grammar tests by language sense 3. Have nothing to do with grammar tests 4. Others_______

13. What's your way to memorize English words?

1. Memorize by rote 2. Enlarge vocabulary by extensive reading 3. Memorize words by word-formation 4. Other ways______

14. What's your opinion on using long sentences in composition?

1. Help to express more complex ideas, I dare to try. 2. Often make mistakes, so I simply use simple sentences. 3. Never consider using attributive clauses or nouns

15. What do you think is the greatest difficulty in your English learning?

1. It's difficult to master the words, which affects the choice. 2. I cannot make a good analysis of sentences and do not comprehensively understand them. 3. Listening. 4. Writing. 5. Others______
